# District Councils and Sanatoria

**Published:** 1906-11-24

**Authors:** 


### DISTRICT COUNCILS AND SANATORIA.

On Monday last the Middlesex District Councils' Association held their annual general meeting at the Guildhall, Westminster, Mr. A. W. Waterlow King, J.P., in the chair.

Mr. E. G. Cole (Wood Green) brought up the question of the provision by the various District Councils and other authorities in the county of proper accommodation for cases of consumption. In his opinion, a sanatorium for dealing with such cases was of really greater importance than a hospital for small-pox patients. For some time past Colonel Clarke had been endeavouring to get various authorities in Middlesex to enter into a scheme for providing a sanatorium, but had only been able to obtain guarantees for twenty-four beds. This was probably because of the large capital cost involved by his proposed scheme. At the present time he understood Colonel Clarke had the offer of a large house in the country, in respect of which he was about to endeavour to evolve a modified scheme. He was most anxious that District Councils in the county should take the matter up. He said he felt sure that the experience which Councils and Boards of Guardians had had during the past two or three years would ensure that there would be no trouble in getting such authorities to take beds for 100 or more, and they would have no trouble in filling them. The idea of Councils and Boards of Guardians working together in the matter was that Boards of Guardians should send Poor-law cases and District Councils should send cases in which the persons were not paupers and did not desire to become such.

There were often young married men with one or two children so suffering. If these cases were taken up, they would not have them or their wives and children becoming paupers in the course of two or three years, but they would save the lives of the men, who could then become good respectable citizens.

It was a most important matter that the question should be taken up. All authorities had not yet decided whether the disease should be made notifiable, but it really was desirable that it should be. He moved that the matter be referred to the Executive Committee of the Association to consider, and if possible to draft a scheme. He did not know of any association so fit as this Association to deal with the matter. There had been too much jealousy in the past—men looking after the interests of their own parish only, and not considering the county as a whole. With this Association, however, they were coming together more and more and getting to see that it was their duty to consider the county as a whole.

Mr. Stanley W. Ball (Willesden), in seconding the resolution, said he thought there were a number of points which must be considered by any committee which dealt with the question. In schemes which had been brought forward enormous sums had been spent on administrative buildings, and it seemed to him that the committee would have to consider whether it would not be possible to acquire land and put up shelters in form of chalets, so that the cost would be exceedingly small. He also considered it would be necessary to formulate some scheme for assisting in the maintenance of the wives and children while the bread-winners were away being cured in a sanatorium. It would be of no use going forward with any scheme which would require very large capital sums to be contributed.

Mr. E. R. Abbott (Ruislip Northwood), Mr. Telling (Hanwell), Mr. B. P. Lascelles (Harrow), and others spoke in favour of a scheme for the provision in the county of accommodation for cases of consumption, and, *inter alia,* reference was made to the terms upon which patients were being admitted to the Mount Vernon Hospital and the possibility of making suitable arrangements for the retention on moderate terms of beds therein.

After full discussion it was decided to call a special meeting of the Association at an early date further to consider the matter, and the Hon. Secretary was directed to obtain as much information on the subject as possible and to invite Colonel Clarke to attend and address the meeting.

